# Time-dependent pH sensing phenomena using CdSe/ZnS quantum dots in EIS structure

**DOI:** 10.1186/1556-276X-9-179

**Published:** 2014-04-12

**Authors:** Pankaj Kumar, Siddheswar Maikap, Amit Prakash, Ta-Chang Tien

**Affiliations:** 1 Thin Film Nano Technology Laboratory, Department of Electronic Engineering, Chang Gung University, Tao-Yuan, Taiwan 333, Taiwan; 2 Bio-Sensor Group, Department of Electronic Engineering, Chang Gung University, Tao-Yuan, Taiwan 333, Taiwan; 3 Material and Chemical Research Laboratories, Industrial Technology Research Institute Hsinchu 310, Taiwan

**Keywords:** pH sensor, CdSe/ZnS quantum dots, EIS structure, Sensitivity

## Abstract

Time-dependent pH sensing phenomena of the core-shell CdSe/ZnS quantum dot (QD) sensors in EIS (electrolyte insulator semiconductor) structure have been investigated for the first time. The quantum dots are immobilized by chaperonin GroEL protein, which are observed by both atomic force microscope and scanning electron microscope. The diameter of one QD is approximately 6.5 nm. The QDs are not oxidized over a long time and core-shell CdSe/ZnS are confirmed by X-ray photon spectroscopy. The sensors are studied for sensing of hydrogen ions concentration in different buffer solutions at broad pH range of 2 to 12. The QD sensors show improved sensitivity (38 to 55 mV/pH) as compared to bare SiO_2_ sensor (36 to 23 mV/pH) with time period of 0 to 24 months, owing to the reduction of defects in the QDs. Therefore, the differential sensitivity of the QD sensors with respect to the bare SiO_2_ sensors is improved from 2 to 32 mV/pH for the time period of 0 to 24 months. After 24 months, the sensitivity of the QD sensors is close to ideal Nernstian response with good linearity of 99.96%. Stability and repeatability of the QD sensors show low drift (10 mV for 10 cycles) as well as small hysteresis characteristics (<10 mV). This QD sensor is very useful for future human disease diagnostics.

## Background

Among the numerous chemical sensors, pH sensor is the major field of research area, which is one of the controlled parameter for the biochemical industrial processes. Lots of aspects have been identified to detect the hydrogen ions under different environment conditions. In development of solid state sensor, recent approaches are ISFET (ion-sensitive field effect transistor), LAPS (light addressable potentiometric sensor), and capacitance-based electrolyte insulator semiconductor (EIS) [1-4]. Among these developments, EIS has shown potential in terms of its simple structure, label-free detection, easy fabrication procedure, and cost effectiveness [5,6]. In addition, nanoparticles have generated considerable interest as diagnostic tool because of their small sizes and comparatively higher surface area that leads to more interaction with ions in solution [7-10]. Semiconductor nanoparticles such as quantum dots (QDs) are one of the major candidates being studied for sensor development [11,12]. The QDs are better than bare SiO_2_ sensing membrane because of their high surface area to volume ratio which gives the platform for controlled immobilization of the biomolecules. In addition, the QDs have been studied as fluorescent labels for bioimaging as well as ionic probes to detect chemical ion concentration in electrolyte solution and immunosensor for cancer detection [13-16]. Long-term environmental stability for robust sensing device is still a major limitation due to environmental factors, such as exposure of reactive ions, humidity, and temperature; results in transformation of nanoparticles such as photooxidation or size change have been reported earlier [17-20]. The controlled distribution of QDs to prevent agglomeration on sensing surface is another important aspect for sensitivity enhancement as well as long-term stability of the device. Some protein-mediated approaches have been demonstrated for the controlled ordering of quantum dots array [21-23]. Chaperonin GroEL protein can be used as template to deposit the quantum dots on sensing membrane as they self-assembled themselves in two-dimensional (2D) array. Although many researchers have reported the sensing properties using different nanoparticles, time-dependent improved pH sensitivity using CdSe/ZnS QDs has not yet been reported.

In this study, time-dependent pH sensing behavior of CdSe/ZnS QD membrane on SiO_2_/Si in EIS structure has been investigated for the first time. The QDs embedded in protein are observed by both atomic force microscope (AFM) and field-emission scanning electron microscope (FE-SEM) images. After annealing at 300°C, the QDs can be observed clearly by SEM due to the removal of protein. The chemical states of the core-shell QDs have been investigated by x-ray photoelectron spectroscopy (XPS). It is found that the QDs are not oxidized, however, water adsorption from environment can be the factor, which results lower defects in the QDs' surface. The values of sensitivity are approximately 34 and 55 mV/pH after initial and 24 months, respectively. The values of differential sensitivity of the QD with respect to bare SiO_2_ sensors are improved from 12 to 32 mV/pH for longer time, owing to higher surface states of the QDs. A good pH sensing linearity of 99.96% is also obtained with QDs-modified sensor.

## Methods

**Figure 1:**
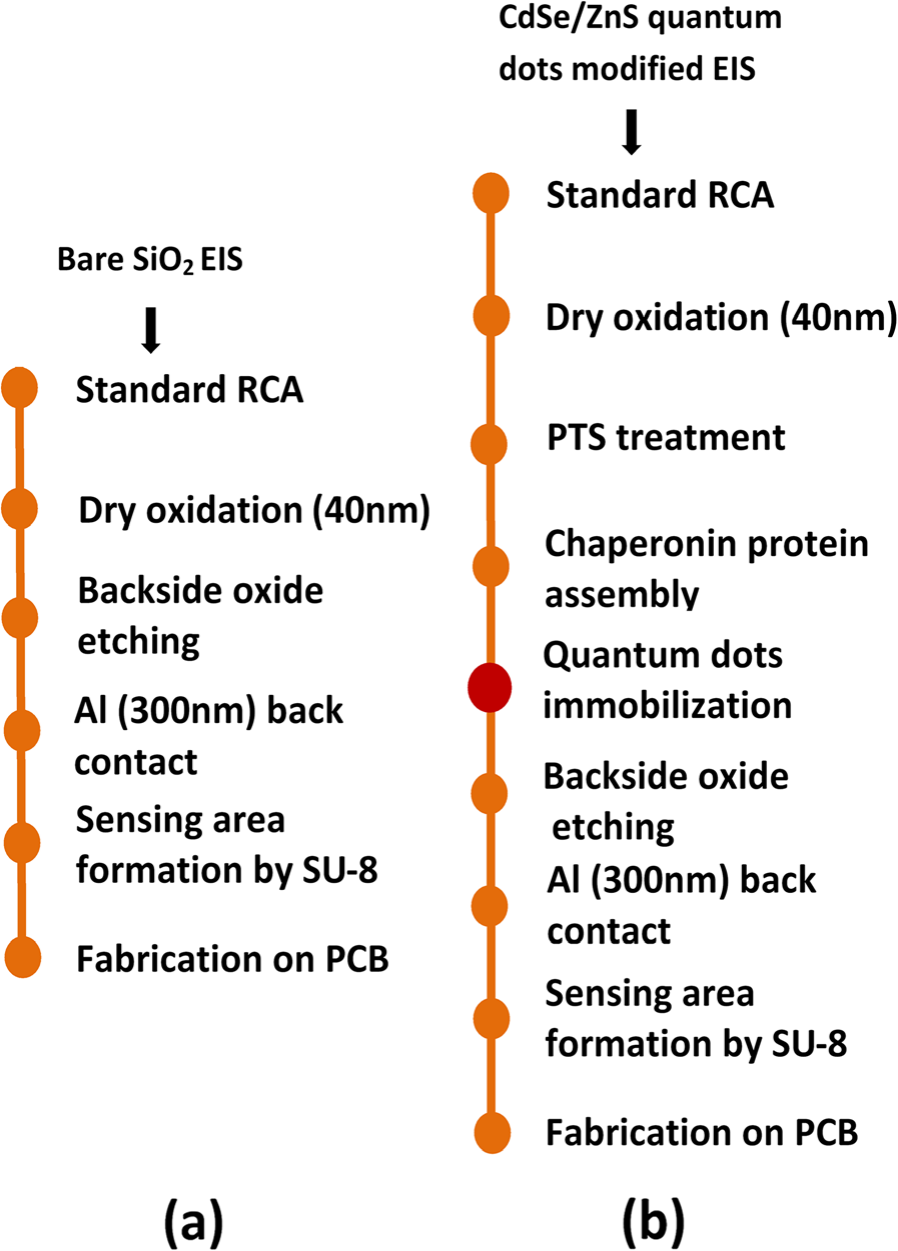
**Fabrication process flow of EIS sensors.** (**a**) Bare SiO_2_. (**b**) CdSe/ZnS quantum dot sensors in the EIS structures.

To study the time-dependent pH sensing behavior of the CdSe/ZnS QDs-modified SiO_2_ surface, a simple EIS structure has been fabricated. The process flow of all the sensors has been shown in Figure 1. A 4-in. Si wafer was cleaned using standard Radio Corporation of America (RCA) procedure. RCA-cleaned wafer was used to grow 40 nm of SiO_2_ layer by dry oxidation process as an insulating layer. Wafers were sonicated in absolute ethanol and dried under nitrogen flow. Dry wafers were used for piranha treatment with temperature maintained at 90°C for 40 min to make - OH-rich surface on SiO_2_ layer. Then wafer samples were rinsed with deionized water and sonicated in spectroscopic grade methanol for 5 min. Samples were dried in oven at 100°C for 60 min. Then, samples were treated with 5% phenyltriethoxysilane (PTS) solution in dry toluene for 60 min under N_2_ flow to further activate the –OH-rich SiO_2_ surface with silane group. After PTS treatment, wafers were rinsed three times with toluene to remove unreacted silane molecules. Further, the samples were rinsed and sonicated in methanol for 1 min and dried at 200°C for 2 h. After cooling, the samples were floated in 0.1 mg/ml chaperonin GroEL protein solution (Takara Bio Inc., Otsu, Shiga, Japan) for 15 min. Then, samples were dried in N_2_ flow and the wafers' surface was treated with the QDs solution (Sigma-Aldrich, St. Louis, MO, USA) for 30 min. The QDs' self-assembly around the protein molecule was expected. Samples were separated out from QD solution, rinsed with toluene three times to remove unbound QDs, and dried under N_2_ flow. Chaperonin GroEL protein consist of 14 oligomeric units which form a cage-like structure with a cavity in its middle. Chaperonin protein molecules can be self-assembled into a crystalline lattice through non-covalent interactions between the proteins. Cysteine amino acids (Cys138) present at the outer side of apical domain and at the bottom of equatorial domain (Cys 458 and Cys 519) have been reported earlier [24]. After CdSe/ZnS QDs distribution over protein array, QDs attached to the chaperonin molecule via ZnS interaction with thiol group of cysteine instead at the central cavity as observed from the microscopic characterization. Chaperonin protein was used for controlling the distribution and immobilization of QDs on SiO_2_ surface. However, this did not play any role in pH sensing. After annealing at 300°C for 30 min in air atmosphere, the protein molecule burned out and the QDs remained on the SiO_2_ surface. This process was optimized and it was repeatable. However, there will be variation of the QD density as well as the sensitivity.

**Figure 2:**
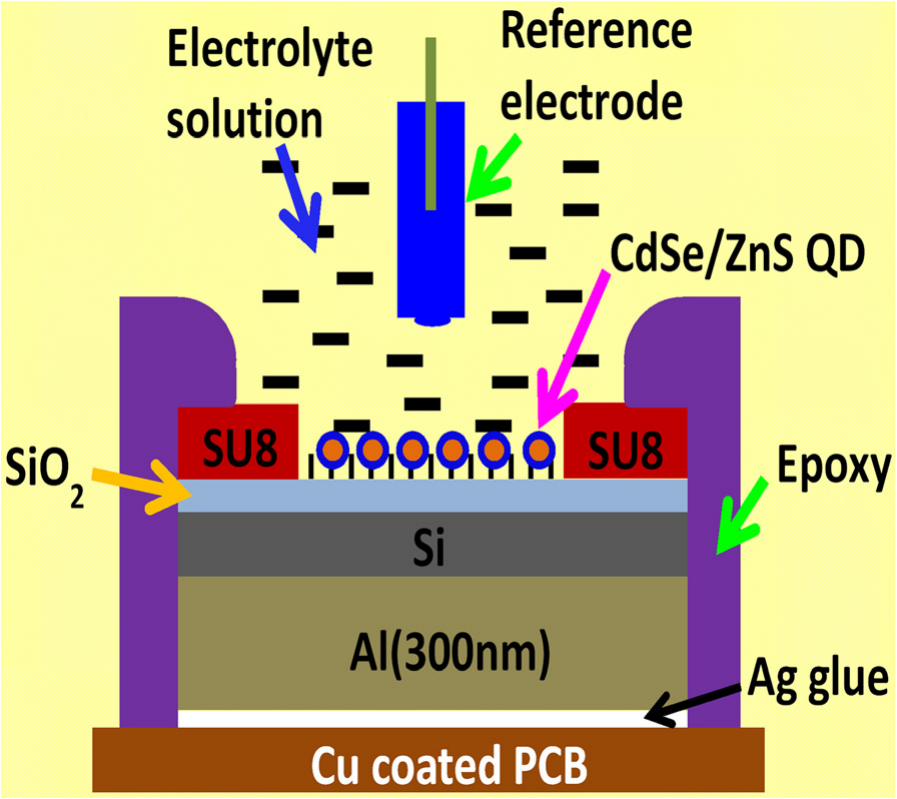
**Schematic diagram of CdSe/ZnS QD sensor in EIS structure on PCB.** The reference electrode and sensor isolation are shown.

To fabricate the device on copper-coated printed circuit board (PCB), the back oxide of Si wafer was etched by BOE (buffer oxide etchant) and the aluminum back electrode was deposited by thermal evaporation. Then, sensing area (3.14 mm^2^) was defined on the device by photolithography using negative photoresist SU-8 (MicroChem, Newton, MA, USA). The device was fixed on the Cu lining pattern on PCB board using silver paste. Finally, an insulating layer of epoxy was used to pack the chip except sensing area. The schematic diagram of the EIS sensor using QDs/SiO_2_ membrane is shown in Figure 2.

The surface topography of chaperonin mediated QDs distribution on SiO_2_ surface was investigated by using an Innova scanning probe microscope (SPM) system (Bruker Corp., Bellerica, MA, USA). The AFM image was measured in tapping mode with a scan at area of 500 × 500 nm^2^. The size and topography of the QDs were investigated using FE-SEM (MSSCORPS Co. Ltd., Taiwan). The chemical bonding of the CdSe and ZnS elements was investigated by XPS. The EIS structure was transferred to the analyzing chamber at ultra-high vacuum of 1 × 10^-9^ Torr. The XPS spectra were recorded using Al K_a_ monochromatic x-ray source with energy of 1,486.6 eV. The scan was from 0 to 1,350 eV with step energy of 1 eV.

Capacitance-voltage (*C*-*V*) measurement was done using HP4284A in different pH buffer solutions. An Ag/AgCl electrode was used as a reference electrode and it was grounded during *C*-*V* measurement. The bias was applied on the Al bottom electrode. All measurements were done at 100 Hz. To obtain the steady results, all samples were kept in reverse osmosis (RO) water for 24 h before measurement. The EIS sensors were washed with deionized (DI) water before electrode transfer to subsequent pH solution. The ConCap response of QD-modified EIS pH sensor was measured up to ten repeated cycles to check any drift in pH sensitivity in each buffer solution. ConCap response was studied from acidic to basic pH and reversed to study the hysteresis effect of EIS sensors. To measure ConCap response, the QD-modified EIS sensor was washed with DI water after each step during repetitive measurement at the same buffer solution.

## Results and discussion

**Figure 3:**
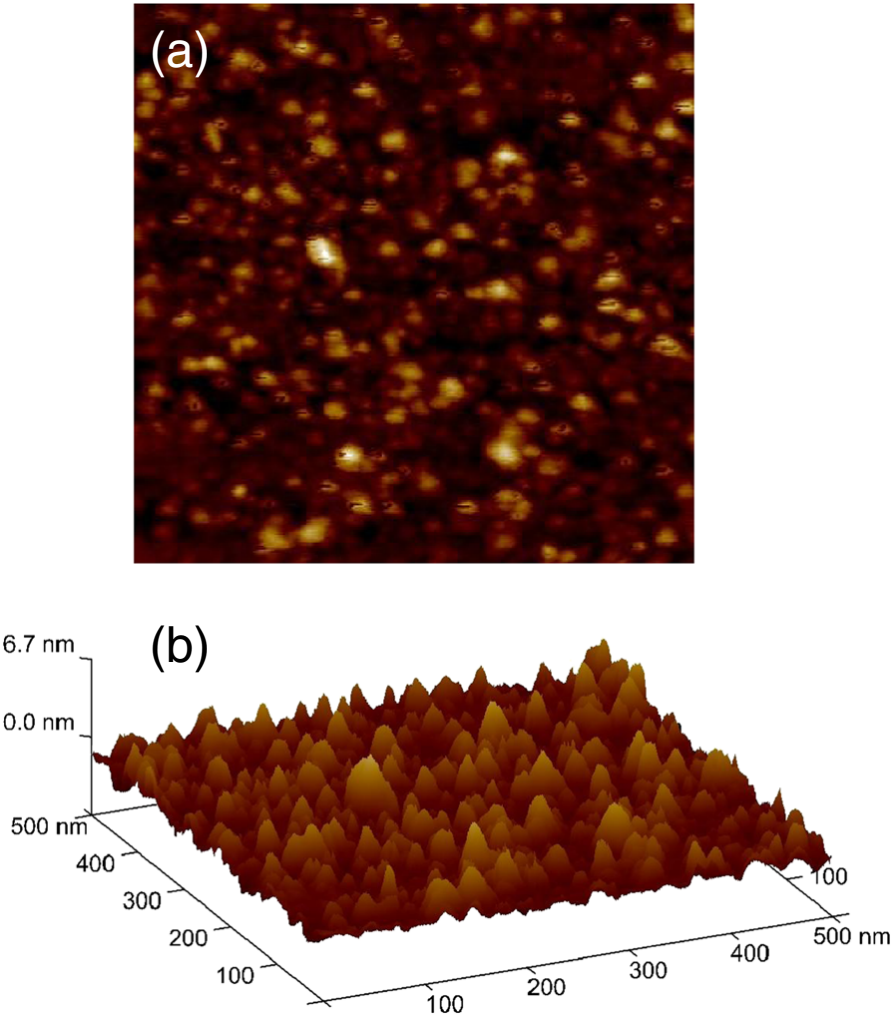
**AFM image of the CdSe/ZnS quantum dots distribution in chaperonin protein on SiO**_**2**_**/Si substrate. (a)** 2D and **(b)** 3D images of quantum dots embedded in protein. The scan area was 500 × 500 nm^2^.

**Figure 4:**
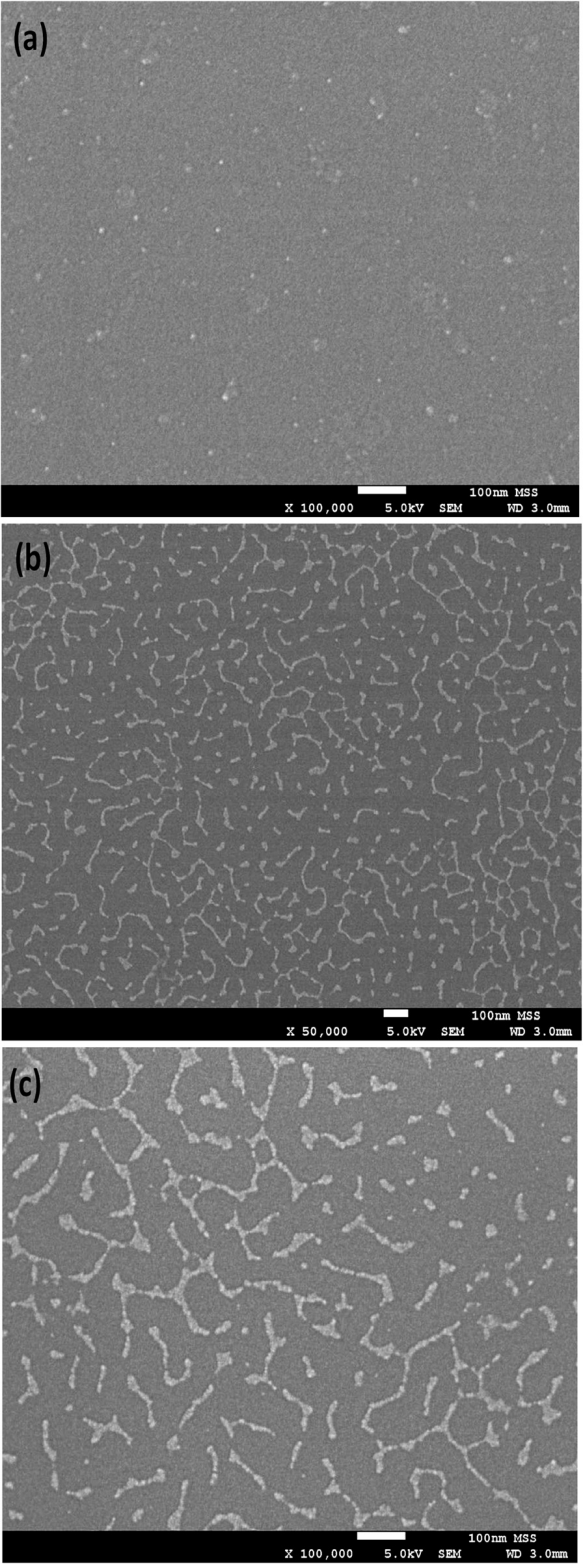
**SEM topography of CdSe/ZnS QDs distribution.** SEM images with **(a)** QDs in protein and after annealing at 300°C for 30 min with different magnifications of **(b)** × 50 and **(c)** × 100 k.

Figure 3 shows topography of the QDs embedded in chaperonin protein, observed by AFM. Two-dimensional AFM image is shown in Figure 3a, and three-dimensional (3D) image is shown in Figure 3b. The average (*R*_a_) and root mean square (rms; *R*_q_) surface roughness are found to be 0.642 and 0.836 nm, respectively. The density of QDs is approximately 10^11^/cm^2^. Quantum dots immobilization and distribution around protein cavity are also observed by FE-SEM, as shown in Figure 4. The distribution of the QDs on chaperonin protein layer attached on SiO_2_ surface (Figure 4a) and very few QDs appear on the surface, as most of the QDs have been attached at both side and the bottom of protein via ZnS-thiol group interaction at cysteine amino acid. After annealing at approximately 300°C, the sacrificial chaperonin protein layer burned out and a structure of quantum dots arranged around the protein molecules developed, as shown by different magnifications in Figure 4b,c. Development of QD ring-like structure after annealing is expected to be due to the removal of sacrificial protein molecules. The diameter of one QD from SEM image is approximately 6.5 nm. The chemical bonding of the QDs has been investigated by XPS, which is discussed below.

**Figure 5:**
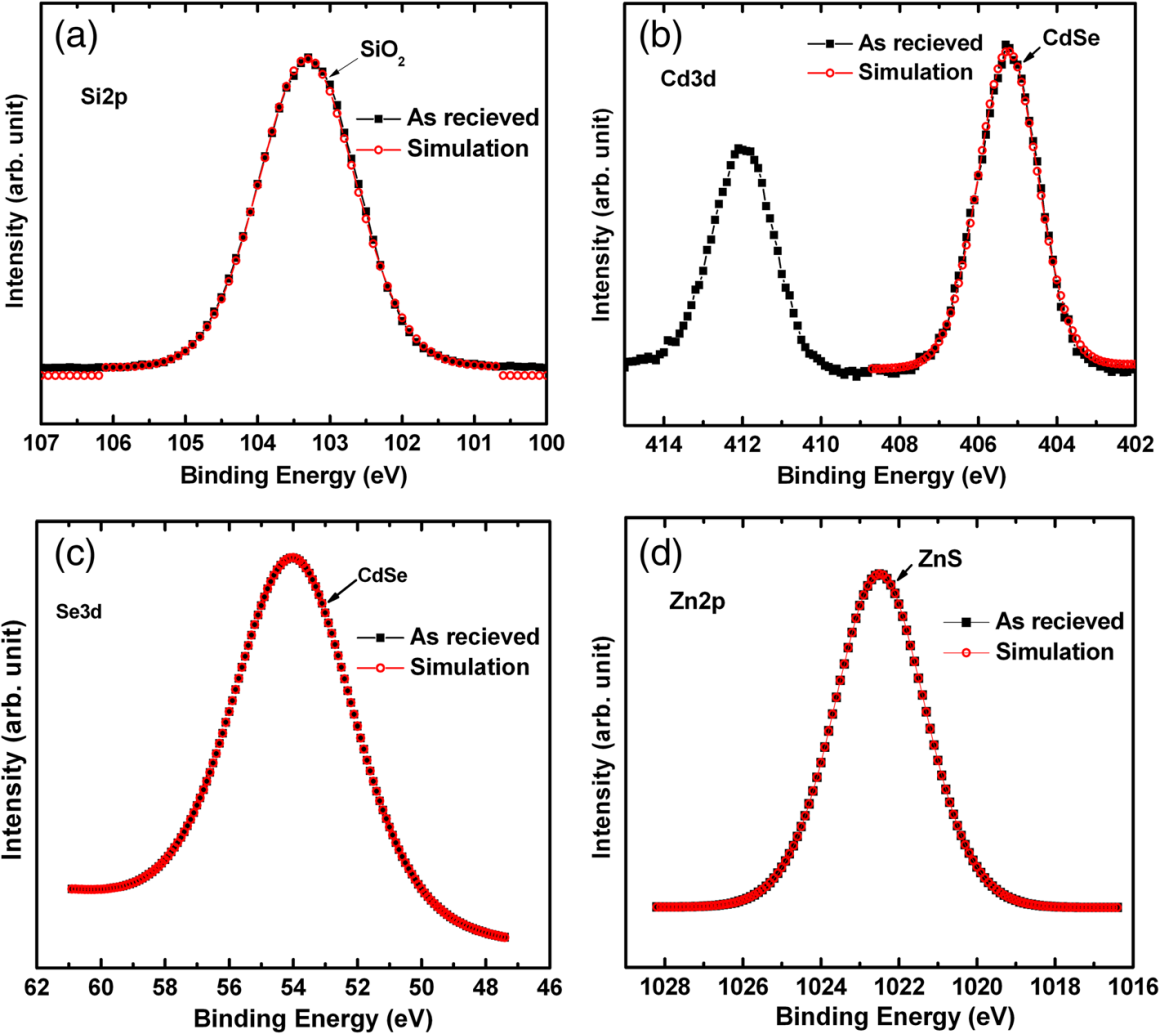
**XPS characteristics of core-shell CdSe/ZnS QDs on SiO**_**2**_**/Si substrate.** Core-level spectra of **(a)** Si2*p* for SiO_2_, **(b)** Cd3*d* for CdSe, **(c)** Se for CdSe, and **(d)** Zn2*p*3 for ZnS are shown. The core-shell CdSe/ZnS QDs are confirmed.

Figure 5 shows the XPS characteristics of bare SiO_2_ and QDs. The peak fitting was performed by Shirley subtraction and Gaussian method. The peak binding energy of Si2*p* is approximately 103.31 eV (Figure 5a), which is similar to the reported value of 103.58 eV [25]. This Si2*p* represents the SiO_2_ film. Figure 5b shows the XPS spectra of 3*d* core-level electrons of the CdSe. The peak binding energies of Cd3*d*_3/2_ and Cd3*d*_5/2_ electrons are found to be 412 and 405.24 eV, respectively. Liu et al. [26] reported the peak binding energy of CdSe at 405.46 eV. The CdSe element is also confirmed by Se fitting with peak energy of 54 eV, as shown in Figure 5c. The core-level energy of Zn2*p*3 is approximately at 1,022.49 eV (Figure 5d), which is close to the reported peak binding energy at 1,022.73 eV [27]. By fitting, ZnS element is confirmed. Therefore, core-shell CdSe/ZnS QDs are confirmed from the XPS analysis. It is important to note that these QDs are not oxidized by environment or our devices' process. It is expected that this QD-modified EIS sensor will have good sensing properties, which are explained below.

**Figure 6:**
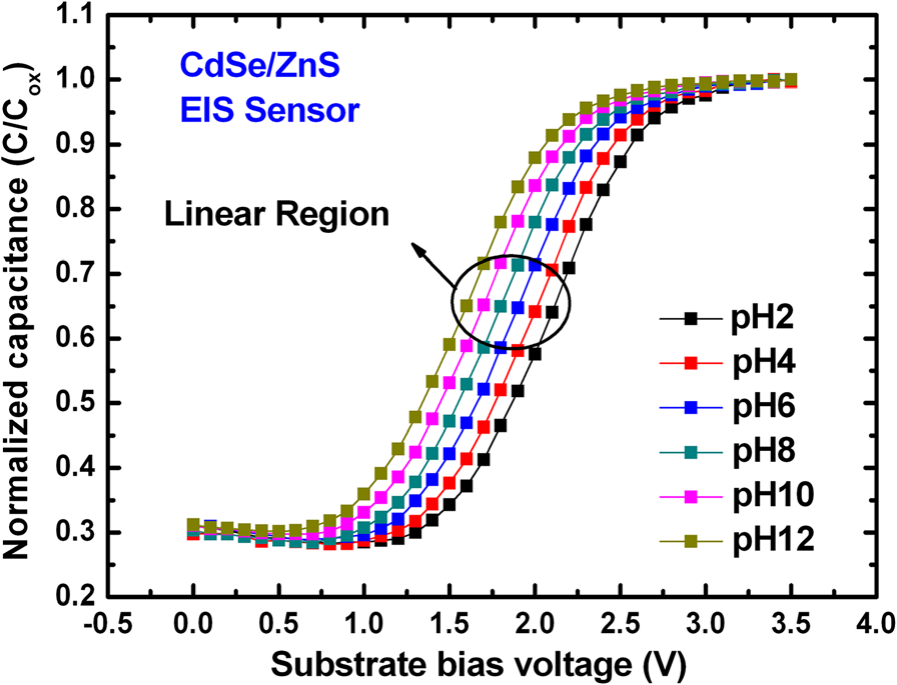
**Typical *C*-*V *characteristics of QD sensor.** The *C*-*V* characteristics with different pH buffer solutions of 2 to 12 are observed after 24 months.

Figure 6 shows *C*-*V* characteristics with different pH buffer solutions for the QD EIS sensor after 24 months. It is noted that higher frequency measurement has lower sensitivity and the lower frequency has a stressing effect on the EIS sensor. That is why the optimized *C*-*V* measurement was done at 100 Hz. The *C*-*V* curves shift, owing to different pH values. The flat band voltage (*V*_fb_) is measured at a normalized capacitance of 0.65. Sensitivity of the sensors is calculated from voltage shift in the *C*-*V* curves with respect to change in pH using the equation as given below:

(1) $$\mathrm{Sensitivity}=\frac{\Delta V_{\mathrm{fb}}}{\mathrm{\Delta pH}}$$

**Figure 7:**
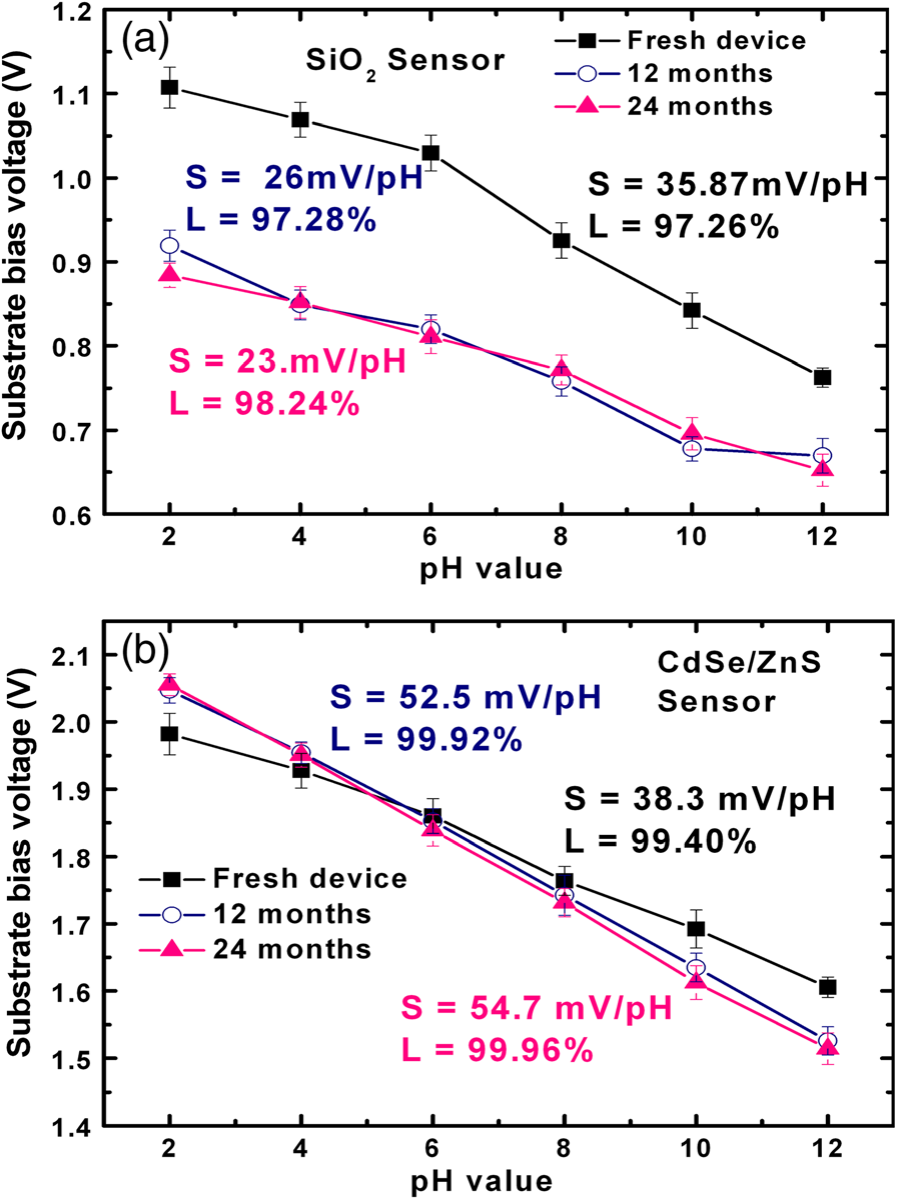
**Time-dependent pH sensitivity.** Sensitivity characteristics of **(a)** bare SiO_2_ and **(b)** CdSe/ZnS QD sensors for 0 to 24 months. Three sensors of each sample are considered to calculate average sensitivity and linearity.

The values of *V*_fb_ decrease with increase in the pH of buffer solutions (Figure 7), which can be explained by the combination of Site Binding model as well as Guloy-Chapman-Stern model at the electrolyte-oxide interface [28]. Bare SiO_2_ sensing membrane at EIS surface undergoes silanol formation in water which further undergoes protonation and de-protonation reaction after contact with electrolyte solution as explained by the Site Binding model.

(2) $$\mathrm{SiOH}+H^{+}↔{\mathrm{SiOH}}_{2}^{+}$$

(3) $$\mathrm{SiOH}↔{\mathrm{SiO}}^{-}+H^{+}$$

According to this model, the combination of ionic states as shown above results from the surface charge at one particular pH. At different pH buffer solutions, the surface charge varies according to the density of ionic states at the oxide surface. However, a collective effect of surface charge and ionic concentration results in the effectively charged layer at sensor-electrolyte interface known as stern layer, which is explained by Guoy-Chapman-Stern model. A combination of surface charge as well as the thickness of electric double layer at sensor-electrolyte interface defines the surface potential of EIS sensor at different pH values. The surface potential of EIS sensing membrane can be determined at particular pH by Nernst equation as shown below:

(4) $$E=E^{0}+2.303\frac{\mathrm{RT}}{F}\mathrm{In}\left[H^{+}\right]$$

where *E* is the sensing membrane potential without electrolyte solution, *R* is the universal gas constant of 8.314 JK^-1^ mol^-1^. *T* is the absolute temperature, and *F* is Faraday constant of 9.648 × 10^-4^C-mol^-1^. It is assumed that the CdSe/ZnS QDs immobilized at SiO_2_ surface have higher negative charge results in the thicker stern layer or more H^+^ ion accumulation at sensor-electrolyte interface results in higher density of ionic states at the surface. The higher ions' reactivity at the sensing membrane surface lead to higher surface potential which ultimately results in more *V*_fb_ shift for the QD sensors.

It is interesting to note that the time-dependent sensitivity of both the EIS sensors is observed over a time period of 24 months. A comparison of the sensitivity and linearity study of bare SiO_2_ and CdSe/ZnS quantum dot sensors at different time periods is shown in Figure 7. Initially, the bare SiO_2_ sensors show the pH sensitivity 35.87 mV/pH with linearity 97.26%. The sensitivity of bare SiO_2_ EIS sensors is not stable and even worse with time (Figure 7a). The values of sensitivities (linearity) are found to be 26 (97.28%) and 23 mV/pH (98.24%) after 12 and 24 months, respectively. The degradation in sensitivity of bare SiO_2_ EIS sensor with time is attributed to the dissolution of silanol at higher acidic or basic pH in electrolyte solution. On the other hand, the sensitivity of the QD sensors shows stable and better response than the bare SiO_2_ sensors. Initially, the CdSe/ZnS QD sensors show the sensitivity of 38.3 mV/pH with good linearity of 99.40% (Figure 7b), which is comparatively higher than the pH sensing response of Au nanoparticles as reported by Gun et al. [10]. The values of sensitivity are improved to 52.5 and 54.7 mV/pH, while the values of linearity are found to be 99.92% and 99.96% after 12 and 24 months, respectively. After 24 months, the sensitivity of the QD sensors is near to ideal Nernstian response. The differential sensitivity of the QD with respect the bare SiO_2_ sensors also remarkably improved from 2 to 32 mV/pH with time. Therefore, the QD sensor can be used as a differential sensor. Cordero et al. [18] proposed the improved luminescence behavior of QDs after passivation of the surface trap states by adsorption of water molecules and reduction in the defect sites at CdSe quantum dots. However, this phenomenon is followed by photooxidation of the QDs' surface, which is opposite of surface passivation, which induces the defects in QDs' surface. In our case, we observe the similar behavior over long time. The passivation of quantum dots' surface by water molecule adsorption is expected from the environment's humidity, as sensor devices were kept at room temperature and measured for pH sensitivity repeatedly. In addition, sensitivity evolution with time is also in agreement of mechanism proposed by Asami et al. [29]. They reported the change in adsorption state of TOPO on CdSe surface as TOPO (Lewis base) passivates the unbonded Se surface on longer photoillumination, and the shift in adsorption state of TOPO leads to the change in surface states of CdSe nanocrystals.

**Figure 8:**
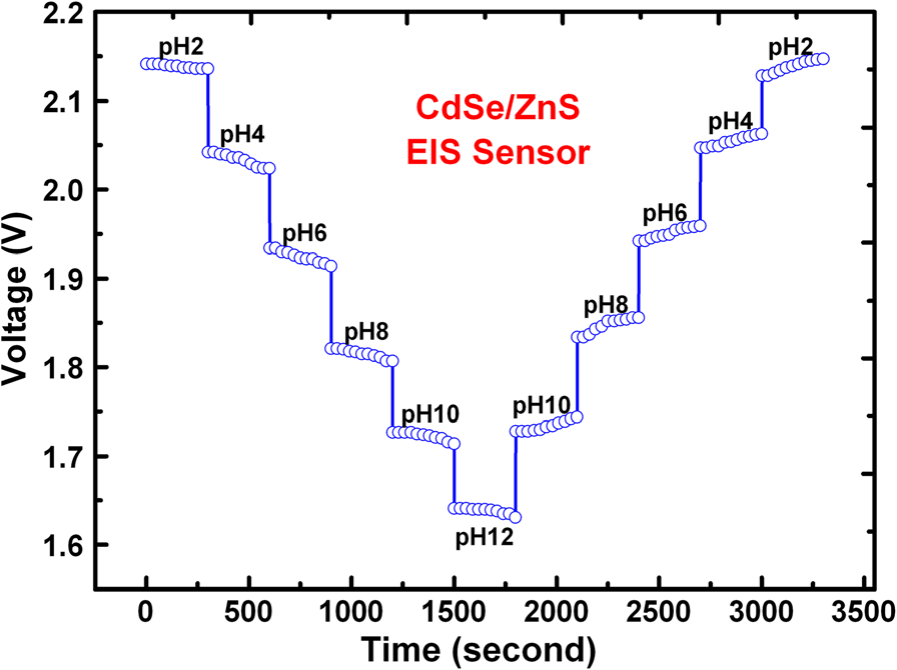
**ConCap response measurements of CdSe/ZnS QD sensors after 24 months.** Ten cycles are performed at each buffer solution with DI water washing of the sensing membrane after every cycle.

Bare SiO_2_ sensor does not respond very well at acidic pH compared to basic pH, while core-shell CdSe/ZnS QD sensor shows good linearity from pH 2 to 12. Bare SiO_2_ shows small pH differentiation for acidic because the isoelectric point of SiO_2_ thin film grown by thermal oxidation is approximately 4.2 [30]. To check the stability and repeatability of fresh bare SiO_2_ and QD EIS sensors, a ConCap response measurement has been studied at 2 to 12 pH buffer solutions, as shown in Figure 8. Bare SiO_2_ sensor shows the comparatively higher drift at highly acidic and highly basic pH due to silanol dissolution in electrolytes (not shown here). The core-shell CdSe/ZnS QD sensor shows acceptable drift of 10 mV as well as small hysteresis (<10 mV) studied up to 10 cycles in each pH buffer solution as well as it shows very less hysteresis effect than the bare SiO_2_ EIS sensors. High surface area as well as sensitivity improvement over the years also suggests that the CdSe/ZnS QD sensor has a potential to detect biomolecules with longer lifetime.

## Conclusions

The CdSe/ZnS QDs in EIS structure have been successfully immobilized on SiO_2_ film using chaperonin protein. The QDs are observed by AFM and FE-SEM images, and the diameter of each QD is found to be approximately 6.5 nm. The core-shell CdSe/ZnS QDs are also confirmed by XPS, and the QDs are not oxidized even after long exposure time in air. Initially, improved pH sensitivity of the QD sensor is observed as compared to the bare SiO_2_ sensor (approximately 38 vs. 36 mV/pH) and it is further improved after 24 months (approximately 55 vs. 23 mV/pH), and the differential sensitivity with respect to bare SiO_2_ sensor is improved from 2 to 32 mV/pH, owing to the reduced defects in QDs with time. Good linearity of 99.96% is also obtained for a longer time. In addition, good stability and repeatability of quantum dots-modified EIS sensors are obtained by ConCap response of devices at 2 to 12 pH buffer solutions. This simple QD EIS sensor paves a way in future human disease investigation.

## Competing interests

The authors declare that they have no competing interests.

## Authors’ contributions

PK fabricated and analyzed the EIS sensors. AP helped to fabricate these sensors also. TCT did XPS characteristics and analysis. This research work was carried out under the instruction of SM. First draft of this manuscript was written by PK. All of the authors revised the manuscript and approved it for publication.
